# HIV-1 interactions with sialic acid-binding bacterial lectins promote virus infectivity *in vitro* and mucosal transmission in humanized mice

**DOI:** 10.3389/fcimb.2026.1879791

**Published:** 2026-09-16

**Authors:** Clauvis Kunkeng Yengo, Xiaomei Liu, Ries J. Langley, Frida Avila, Manish Sagar, Christina Ochsenbauer, Barbara A. Bensing, Catarina E. Hioe

**Affiliations:** 1 Division of Infectious Diseases, Department of Medicine, Icahn School of Medicine at Mount Sinai, New York, NY, United States; 2 Research and Development Service, James J. Peters VA Medical Center, Bronx, NY, United States; 3 Molecular Medicine and Pathology, University of Auckland, Auckland, New Zealand; 4 Maurice Wilkins Centre for Molecular Biodiscovery, University of Auckland, Auckland, New Zealand; 5 Department of Medicine, Boston University Chobanian & Avedisian School of Medicine, Boston, MA, United States; 6 Department of Virology, Immunology and Microbiology, Boston University Chobanian & Avedisian School of Medicine, Boston, MA, United States; 7 Department of Medicine, Heersink School of Medicine, University of Alabama at Birmingham, Birmingham, AL, United States; 8 Department of Medicine, San Francisco Veterans Affairs Medical Center and University of California, San Francisco, San Francisco, CA, United States

**Keywords:** HIV-1, bacterial lectins, mucosal transmission, sialoglycan, trans-infection

## Abstract

Most human immunodeficiency virus type 1 (HIV-1) transmission occurs at mucosal surfaces, which are colonized by the host microbiota. However, interactions between HIV and bacteria or bacterial products derived from the human microbiome are poorly characterized, and their biological consequences are largely unexplored. Here, we evaluated the effects of sialic acid-binding lectins expressed by bacterial species ubiquitous in the human microbiota on HIV-1 infectivity using viruses produced in 293T cells and human primary cells. We demonstrated that these bacterial lectins enhanced HIV-1 infectivity in a sialoglycan-dependent manner. Specifically, Siglec-like binding region lectins (SLBR-N, SLBR-H, and SLBR-B) from *Streptococcus gordonii* and staphylococcal superantigen-like lectins (SSL3, SSL4, and SSL11) from *Staphylococcus aureus* increased HIV-1 infectivity to varying extents, depending on lectin type and virus strain. Among these lectins, SLBR-N exhibited the greatest potency, corresponding with its superior ability to bind virions and promote virus–cell attachment. This enhancing activity was observed for direct infection of TZM-bl reporter cells and primary CD4^+^ T cells, as well as trans-infection in the presence or absence of the mannose-binding host lectin DC-SIGN. Importantly, these findings were corroborated *in vivo* using humanized mice, in which pre-exposure to SLBR-N promoted rectal HIV-1 transmission and increased viral burdens in plasma and splenic cells. Collectively, the data show sialoglycan-binding bacterial lectins as microbial factors that can enhance HIV-1 transmission at mucosal surfaces, highlighting a potential direct role for the microbiota in modulating HIV-1 acquisition risk.

## Introduction

Most human immunodeficiency virus type 1 (HIV-1) transmission occurs through the mucosa during sexual contact, perinatal events, and breastfeeding (Gonzalez et al., 2019; Tebit et al., 2012). At the mucosal surfaces, the virus interacts not only with host cells but also with bacteria in the microbiota. Indeed, bacterial dysbiosis is linked to increased risk of HIV-1 acquisition (Li et al., 2023, 2025; Liu et al., 2017; Zilberman-Schapira et al., 2016), although this has been attributed mainly to the associated inflammation and compromised mucosal barrier.

Mucosal microbial communities are dynamic and modulated by hormonal changes and contraceptive use, among other factors (Balle et al., 2023; Carson et al., 2021; Noel-Romas et al., 2020; Ratten et al., 2021; Song et al., 2020; Vinerbi et al., 2025). Depot-medroxyprogesterone acetate (DMPA or Depo-Provera), an injectable contraceptive that has been associated with increased HIV-1 acquisition risk (Brind et al., 2015; Hapgood et al., 2018; Morrison et al., 2015; Polis et al., 2016; Ralph et al., 2015), induces shifts in the vaginal microbiota, signified by reduced *Lactobacillus* dominance, enrichment of vaginosis-associated bacteria such as *Prevotella*, and retention of a few community networks that include *Streptococcus* and *Mycoplasma* (Brooks et al., 2017; Roxby et al., 2016; Yang et al., 2019). Despite these associations, direct mechanistic links between microbiome changes and HIV-1 transmission remain poorly defined.

Among vaginosis-associated bacteria, *Prevotella timonensis* encodes mucus-degrading enzymes, including fucosidases and neuraminidases (sialidases) that degrade mucin O-glycans, which are critical for maintaining healthy cervicovaginal barriers (Pelayo et al., 2024; Segui-Perez et al., 2024). *P. timonensis* also enhances HIV-1 infection in vaginal CD4^+^ T cells and promotes trans-infection from CD1c^+^ dendritic cells and vaginal Langerhans cells to CD4^+^ target cells (van Smoorenburg et al., 2025; van Teijlingen et al., 2022, 2024). Notably, these effects are driven by bacteria–cell interactions (van Teijlingen et al., 2020), rather than direct bacteria–virus engagement.

Investigations on direct HIV-1–bacteria interactions have been limited, although intimate connections between bacteria and other viruses have been well documented. Human norovirus binds the histo-blood group antigen (HBGA) glycans from commensal *Enterobacter cloacae* to infect B cells (Jones et al., 2014; Karst, 2016; Miura et al., 2013). Poliovirus binds bacterial lipopolysaccharide (LPS) via its capsid, increasing virus thermostability, resistance to bleach, and virus replication in the intestine (Kuss et al., 2011; Robinson et al., 2014). Similarly, murine mammary tumor virus (MMTV) binds bacterial LPS via LPS-binding host proteins (CD14, MD2, and TLR4) incorporated in the virus envelope (Wilks et al., 2015), and MMTV-bound LPS triggers TLR4 to induce interleukin-6 (IL-6) and IL-10, thereby creating an immunosuppressive environment that permits virus persistence (Jude et al., 2003; Kane et al., 2011).

At the mucosal sites such as the urogenital tract, oral cavity, and gastrointestinal tract, commensal and pathobiont bacteria express a variety of lectins that recognize glycans on host cells, mucus, and extracellular matrix proteins, and play key roles in bacteria adhesion, biofilm formation, and immune evasion (Fares et al., 2025). We previously revealed that bacterial lectins also interact with HIV-1 virions and the virus envelope glycoprotein (Env) in a glycan-dependent manner, resulting in distinct outcomes depending on the lectins. HIV-1 binding to sialylated O-glycan-specific Siglec-like binding region (SLBR) lectins from *Streptococcus gordonii* resulted in enhanced virus infectivity, whereas the interaction with high-mannose N-glycan-specific FimH lectin from *Escherichia coli* caused no changes (Heindel et al., 2024), implicating the role of specific bacterial lectins as modulators of HIV-1 infection. However, the previous study was limited to these lectins and a few virus strains, and the biologic consequences *in vivo* were unexplored.

The present study investigated sialoglycan-binding lectins from *S. gordonii* and *Staphylococcus aureus* for the ability to augment infection across HIV-1 strains, including viruses produced in primary human CD4 T cells. *S. gordonii* is a commensal bacterial species that is found in the oral cavity, colonizing tooth surfaces beginning in infancy and is linked to tooth decay and endocarditis (Bensing et al., 2018; Mosailova et al., 2019; Park et al., 2020). In addition, *S. gordonii* can also be found in the skin, upper respiratory tract, and intestine (Park et al., 2020). *S. aureus* is a pathobiont that colonizes humans soon after birth. *S. aureus* is ubiquitous, present in 30%–50% of the human population, including both healthy and hospitalized individuals, with reservoirs in the nasopharynx, the skin, the genital tract, and the intestinal tract (Acton et al., 2009; Bhalla et al., 2007; Squier et al., 2002). Glycan-dependent HIV-1 interactions with these bacterial lectins were ascertained using a glycan competition assay and lectin mutants. In addition to direct infection, we evaluated the effect of bacterial lectins on HIV-1 trans-infection in the presence and absence of the host lectin, DC-SIGN. Finally, we assessed lectin activity on rectal HIV-1 infection using a humanized mouse model. These findings identify sialic acid-binding lectins produced by common bacteria of the human microbiota as potential modulators of HIV-1 transmission.

## Materials and methods

### Ethics statement

Animal work was reviewed and approved by the Icahn School of Medicine at Mount Sinai Institutional Animal Care and Use Committee.

### Cells, lectins, and viruses

HEK293T cells were purchased from the American Type Culture Collection (ATCC). The following reagent was obtained through BEI Resources, NIAID, NIH: TZM-bl cells, HRP-8129, contributed by Dr. John C. Kappes, Dr. Xiaoyun Wu, and Tranzyme Inc (Platt et al., 1998). The following reagent was obtained through the NIH HIV Reagent Program, Division of AIDS, NIAID, NIH: parental Raji cells (Raji-0), ARP-9944, and DC-SIGN-expressing Raji cells (DC-SIGN+ Raji), ARP-9945, contributed by Dr. Li Wu and Dr. Vineet N. KewalRamani. These cell lines were maintained in Dulbecco’s modified Eagle medium (DMEM, Lonza) supplemented with 10% heat-inactivated fetal bovine serum (FBS), penicillin/streptomycin (100 U/mL), and L-glutamine.

Peripheral blood mononuclear cells (PBMCs) were isolated from Leukopaks purchased from the New York Blood Center, and CD4^+^ T cells were purified from human peripheral blood using the MagCellect Human CD4^+^ T cell isolation kit (R&D Systems). Primary cells were maintained in complete RPMI 1640 medium supplemented with 10% FBS, 100 U/mL penicillin, 100 μg/mL streptomycin, and 2 mM L-glutamine.

SLBRs were expressed in *E. coli* transformed with the corresponding plasmids and purified as previously described (Bensing et al., 2016; Heindel et al., 2024); only wild-type SLBRs were evaluated in this study. Wild-type and mutated staphylococcal superantigen-like (SSL) lectins were expressed in *E. coli* and purified as previously described (Hermans et al., 2012). Each lectin preparation was subjected to endotoxin removal and tested for its effect on reporter β-galactosidase activity in TZM-bl cells before use in the experiments.

Proviral infectious molecular clone (IMC) plasmids pNL-sNluc.6ATRi-B.JR-FL.ecto/K4760 and pNL-sNluc.6ATRi-C.1086.B2.ecto/K4830 encode *env* sequences of HIV-1 strains B.JRFL and C.1086.B2, respectively, and are engineered to express the secreted Nano-Luciferase (sNLuc) reporter (Gornalusse et al., 2021; Indihar et al., 2024). The IMC plasmid of B.JRFL was obtained from Dr. Jerome A. Zack (UCLA) (Roth et al., 2002). The following reagents were obtained through BEI Resources, NIAID, NIH: HIV-1 IMC, pREJO.c/2864, HRP-11746: pREJO.c/2864, contributed by Drs. John Kappes and Christina Ochsenbauer (Ochsenbauer et al., 2012). These IMCs were generated by transfecting HEK293T cells using JetPRIME (Polyplus). Viral supernatants were filtered (0.45 μm) and pelleted through a 20% sucrose cushion by ultracentrifugation to enrich for virions while minimizing glycoconjugates from serum and cellular sources. Viral pellets were resuspended in PBS, titrated on TZM-bl cells, aliquoted, and stored at −80 °C.

Replication-competent HIV-1 clones 1239-8, 1239-9, 1924-H5, 1924-D12, 4102-3, 4102-61, 4102-80, and 4102-68 were produced in transfected 293T cells and then passaged on primary CD4 T cells (Registre et al., 2020).

Virus treatment with neuraminidase was performed at 37°C for 1 h using neuraminidases from *Clostridium perfringens* (5 units/mL) or *Arthrobacter ureafaciens* (10 units/mL), both from Sigma.

### Virus infectivity assay

Virus infectivity in TZM-bl target cells was performed as described in Heindel et al. (2024), except that DEAE-Dextran was excluded and Beta-Glo^®^ Assay System (Promega) reagents were used for detecting virus infection. Infection in primary phytohemagglutinin P (PHA-P)-activated peripheral mononuclear cells or CD4^+^ T cells was measured using the Nano-Glo^®^ Luciferase Assay System (Promega). For these assays, virions were pre-incubated with titrated amounts of lectins for 1 h at 37 °C prior to the addition of target cells (5,000 cells/well) and incubated for 48 h at 37 °C. Each condition was tested in duplicates or triplicates. Assay controls included replicates of target cells alone (cell control), target cells with virus alone (virus control), and target cells with lectin alone (lectin control). To test the effects of sialyated O-glycan-rich mucin (bovine submaxillary gland mucin, Thermo Fisher Scientific, Cat # J63859MC) versus high-mannose-bearing mannan (Sigma-Aldrich, Cat # M7504), lectin was pretreated with each of these reagents (1 mg/mL) and then incubated with virus.

### SLBR lectin binding to virions by glutathione beads

Virions [1 × 10^8^ viral RNA (vRNA) copies/mL] were incubated with glutathione *S*-transferase (GST)-tagged SLBR lectin (0.4 µM each) or left untreated, for 1 h at 37 °C. Mucin-treated lectin was also tested to assess glycan-specific effect. Pierce™ Glutathione Magnetic Agarose beads (100 µL; Thermo Fisher Scientific) were then added and incubated for 1 h at room temperature. Beads were pelleted, washed three times with PBS to remove unbound virus, and quantified for bead-associated vRNA by RT-qPCR using the QIAamp Viral RNA Mini kit (Qiagen, Cat # 52904). cDNA was synthesized using the High-Capacity RNA to cDNA Kit (Thermo Fisher Scientific, Cat # 4387406) and quantified by real-time PCR using the ABI PRISM 7900HT system. The assay was also performed after virus–lectin incubation for 24 h at 37°C and yielded comparable results (data not shown).

### Virus attachment to cells in the presence of lectin

Virions were pre-incubated with lectins at 37 °C for 1 h, then added to TZM-bl or 293T cells (1 × 10^6^ cells) on ice and maintained for another 1 h to allow binding; cells were then washed three times to remove unbound virions and lectins. vRNA on cells was quantified using RT-qPCR as described above.

### SSL lectin binding to virions on Luminex beads

SSL lectin binding to virions was quantified using a Luminex multiplex bead assay. Sucrose cushion-purified pre-titrated virions were covalently coupled to Luminex beads with the XMAP antibody coupling kit and incubated with titrated amounts of His-tagged SSL wt and mutant. Lectins bound to virions were detected using biotinylated anti-His antibodies (Abcam), followed by PE-labeled streptavidin (Abcam). Data were acquired with a Luminex Bio-Plex FlexMAP 3D system.

### Trans-infection

Virus trans-infection assay was performed as described in Jan et al. (2019). JRFL virions were pre-incubated with titrated amounts of SLBR-N for 1 h at 37 °C. Cells (HEK293T, Raji-0, RAJI-DC-SIGN) were added and incubated for an additional 1 h at 37 °C. After washing three times with culture medium to remove unbound virus, cells were co-cultured with TZM-bl reporter cells at a 1:1 ratio for 48 h at 37 °C. Infection of the TZM-bl cells was quantified using a luminescence-based β-galactosidase assay (Beta-Glo, Promega). For trans-infection assay with PHA-P-stimulated PBMCs, sNLuc JRFL virions were used and infection was measured with the Nano-Glo^®^ Luciferase Assay System.

### Humanized mouse experiments

Humanized mice were purchased from the Columbia University Humanized Mouse Core (CUHMC, New York, NY). These mice were generated as previously described (Li et al., 2017). Briefly, 6- to 8-week-old female NSG mice received sublethal total body irradiation (1 Gy) using an x-ray irradiator (RS-2000, Rad Source Technologies, Suwanee, GA), followed by surgical implantation of a 1-mm³ human fetal thymic fragment under the kidney capsule. Mice also received intravenously human fetal liver hematopoietic stem cells (HSCs, 1 × 10^5^ cells) purified using a Human CD34 MicroBead Kit (Miltenyi Biotec, Cologne, Germany). Peripheral blood samples were collected by submandibular bleeding at 8 and 12 weeks post-transplantation, and human hematopoietic chimerism was analyzed by flow cytometry using fluorochrome-conjugated monoclonal antibodies (anti-mouse CD45-FITC and Ter119-PerCP-Cy5.5), as well as anti-human CD45-BV605, CD3-AF700, CD4-BV786, CD8-PE/Dazzle594, CD19-PE, and CD14-APC/Cy7 (all from BioLegend, San Diego, CA). Flow cytometric analysis was performed using an LSRFortessa (BD Biosciences, San Jose, CA), and data were analyzed with FlowJo software (Tree Star, Ashland, OR).

Mice with >20% human CD45^+^ leucocytes reconstitution were selected for experiments and randomly assigned into two groups (*n* = 5/group). Mice were rectally inoculated with increasing doses of JRFL IMC [350, 700, or 1,400 TCID_50_ (50% tissue culture infectious dose)] administered either with or without SLBR-N (0.06 μM in 10 μL/animal). Blood was collected periodically for measurement of plasma viral load.

### Measurement of plasma vRNA and cell-associated vRNA

vRNA was extracted from plasma using the QIAamp viral RNA Mini kit, while total RNA from blood and tissue was extracted using the Qiagen RNeasy Mini kit. cDNA was prepared using a High-Capacity cDNA Reverse Transcription kit with RNase Inhibitor (Thermo Fisher Scientific, Cat # 4374966) on a C1000 Touch Thermal Cycler. HIV cDNA was then quantified by real-time PCR using a Taqman-based assay on an ABI 7500 Fast Real Time PCR System (Applied Biosystems) as previously described (Hioe et al., 2022). The HIV *gag* sequences of the forward and reverse primers and the Taqman probe were 5’-CAG CCA AAA CTC TTG CTT TAT GG-3’, 5’-GGG ACC AGC AGC TAC ACT AGAA-3’, and NDP:5’-6FAM TGA TGA CAG CAT GCC AGG GAG TGG MGBNFQ-3’, respectively. Cell-associated vRNA was measured simultaneously with cell-derived GAPDH RNA, which was quantified using a Pre-developed Taqman assay reagents kit (Applied Biosystems, Cat # 4333746F).

### Statistical analyses

Data analyses were performed using GraphPad Prism software with statistical tests specified in figure legends.

## Results

### Streptococcal SLBR lectin-mediated enhancement of HIV-1 infectivity varies by viral strain and lectin specificity

We previously showed that SLBR lectins from *S. gordonii* enhanced virus infectivity through interactions with glycan structures on HIV virions (Heindel et al., 2024). However, the previous experiments were performed in the presence of a polycation, diethylaminoethyl (DEAE) dextran, which augments HIV infection by countering the electrostatic-repulsive forces between virus and cell surface (Denning et al., 2013; Ozaki et al., 2012; Platt et al., 2010). Under those conditions, only modest two- to fourfold increases in infectivity were observed (Heindel et al., 2024). To isolate the effects of bacterial lectins, DEAE dextran was excluded from experiments in the present study.

In addition to SLBR lectins, we also evaluated SSL lectins from *S. aureus*. Although both SLBR and SSL lectins recognize terminal sialic acids, they exhibit distinct glycan preferences (**Figure 1**). SLBR-N preferentially binds terminal α2,3 sialic acids present in 3′ sialyllactosamine (sLn) and sialyl Lewis X (sLe^X^) on core 2 *O*-glycans (Di Carluccio et al., 2024; Walker et al., 2022). SLBR-B recognizes sialyl T-antigen on core 1 *O*-glycans, whereas SLBR-H has broader specificity, binding sLn and other sialoglycans on both core 1 and core 2 *O*-glycans (Bensing et al., 2016, 2018, 2022; Deng et al., 2014). SSL lectins similarly recognize the minimal core ligand sLn but show greater affinity for sLe^X^ (Chung et al., 2007; Hermans et al., 2012; Koymans et al., 2015, 2017), although each has a preference for terminal α2,3 sialic acids on distinct N-linked or O-linked oligosaccharides (Consortium for Functional Glycomics glycan array database SSL3: ID 1000985, SSL4: ID 1000986, and SSL11: ID 1000754).

**Figure 1:**
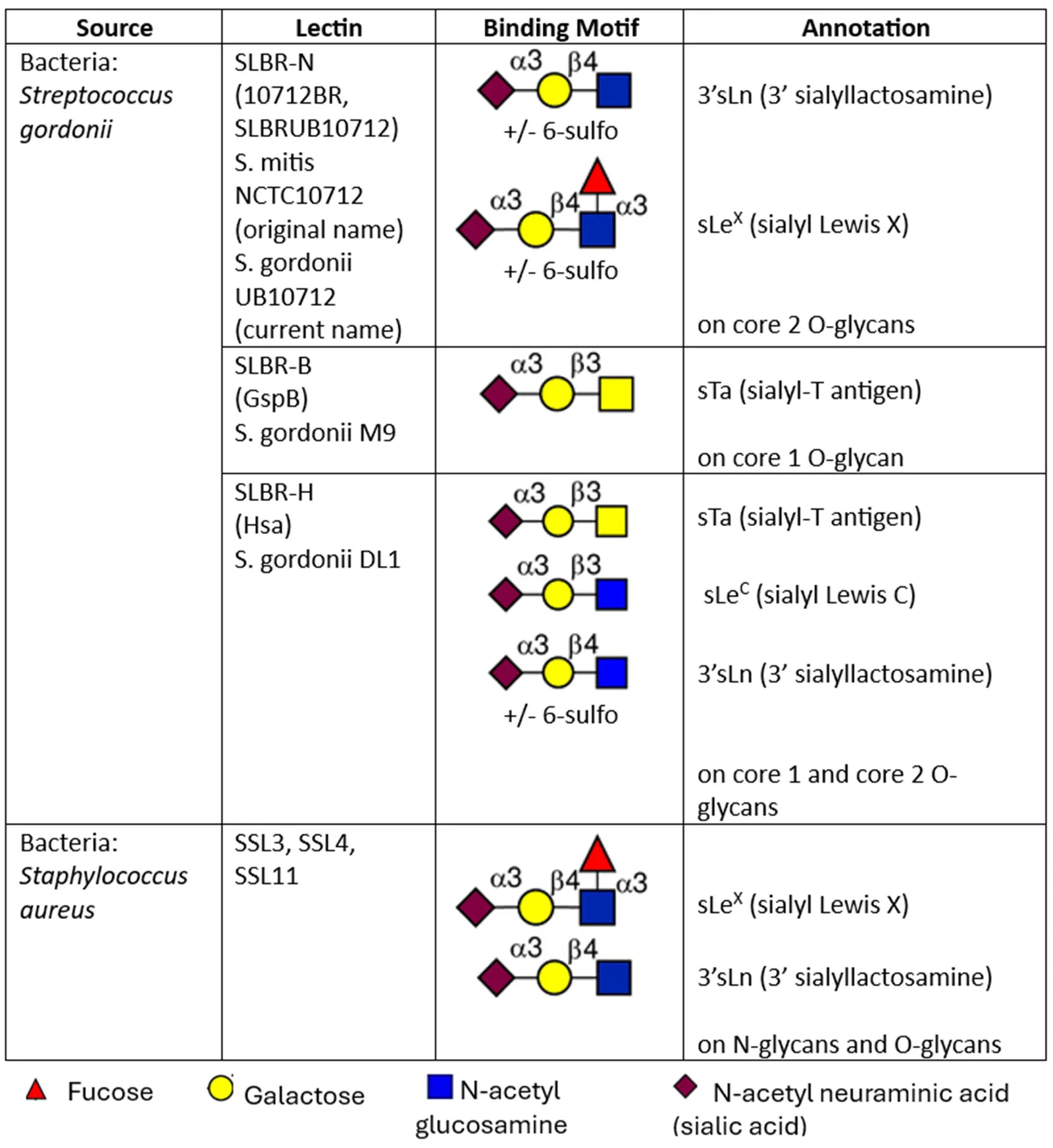
Glycan specificities of SLBRs and SSLs as previously reported. Glycan specificities for SLBRs were determined using glycan microarrays and enzyme-linked binding assays (Di Carluccio et al., 2024; Bensing et al., 2016; Bensing et al., 2022; Deng et al., 2014), whereas those of SSLs were defined by glycan microarrays, surface plasmon resonance, and X-ray crystallography (Consortium for Functional Glycomics glycan array database; Chung et al., 2007; Hermans et al., 2012; Koymans et al., 2015; Koymans et al., 2017).

To assess SLBR-mediated enhancement of HIV infectivity, we initially tested HIV-1 IMCs JRFL (chronic) and REJO.c (transmitter/founder) (Ochsenbauer et al., 2012), produced from 293T cells. IMC virions were pre-incubated with titrated concentrations of individual lectins at 37°C for 1 h and then added to TZM-bl reporter cells. Infectivity was quantified after 48 h by measuring β-galactosidase activity using a chemiluminescent substrate, with relative luminescence units (RLU) as the readout. Because the RLU values of untreated virus controls varied among virus strains and between independent experiments, infectivity is presented as the fold changes in RLU relative to the corresponding untreated virus control to facilitate comparison across viruses and experiments.

Lectin treatment enhanced virus infectivity in a dose-dependent manner, with the magnitude of enhancement varying by both viral strain and lectin (**Figure 2A**). Addition of the lectins alone to TZM-bl cells did not alter reporter β-galactosidase activity. Comparable results were observed whether the lectins were pre-incubated with virus or added simultaneously to virus and cells (**Supplementary Figure 1**). In contrast, we previously showed that SLBR-N had no effect when it was added to TZM-bl target cells before the addition of virus or after infection, indicating that this lectin exerts its activity on virions and not on target cells (Heindel et al., 2024). Notably, SLBR-N produced greater enhancement than SLBR-H or SLBR-B for both JRFL and REJO.c (**Figure 2A**). These results are also consistent with our prior findings using other HIV-1 strains, including clade C Z331M and CRF01_AE CMU06 (Heindel et al., 2024).

**Figure 2:**
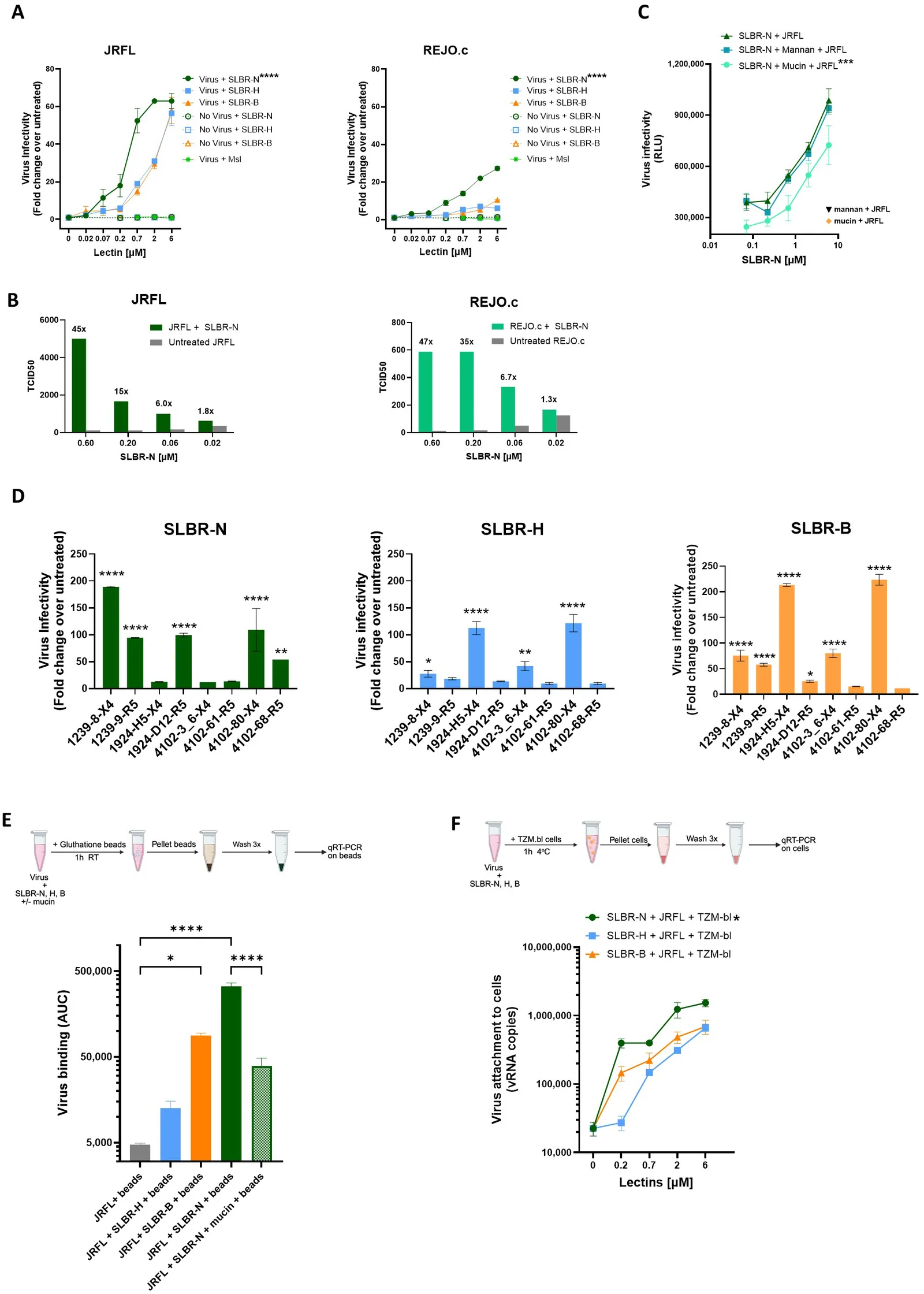
SLBR-N, SLBR-H, and SLBR-B enhance virus infectivity to varying levels across HIV-1 strains. **(A)** Effects of SLBR lectins on the infectivity of HIV-1 virions produced in 293T cells. JRFL and REJO.c IMC virions were pre-incubated with titrated amounts of lectins at 37 °C for 1 hour, and infectivity was quantified in TZM-bl reporter cells by beta-galactosidase activity using a chemiluminescent substrate after 48 hours. For comparison, the effects of the control lectin Msl were assessed in parallel. The effects of lectins alone, without virus, on TZM-bl cells were also measured. Infection with untreated JRFL and REJO.c yielded mean RLU values of 8,899 and 15,973, respectively, whereas background signals from uninfected cells were 4,164 and 4,172 RLU. To facilitate comparison across virus strains, infectivity is presented as fold changes relative to untreated virus, and data are shown as mean ± SD. RLU: relative luminescence unit. **** p<0.001 for SLBR-N vs. SLBR-H and SLBR-B by two- way ANOVA with Šídák's multiple comparison. **(B)** Enhancing effect of SLBR-N on HIV-1 infectivity measured by TCID50. JRFL and REJO.c IMC virions were serially diluted and incubated with or without SLBR-N (0.6-0.02 μM) for 1 hour at 37°C. The reciprocal virus dilutions required to reach 50% infection (TCID50) were calculated in GraphPad Prism using non-linear regression. TCID50 values for SLBR-N-treated virus at each lectin concentration and for untreated virus are shown, together with the fold increases relative to the untreated control. **(C)** Enhancing activity of SLBR-N was decreased by O-glycan-bearing mucin but not high-mannose N- glycan-bearing mannan. SLBR-N was pre-incubated with 1 mg/mL mucin or mannan at 37 °C for 1 hour, followed by incubation with JRFL virions for an additional 1 hour at 37 °C and then addition of TZM-bl cells. Virus infectivity was determined by measuring beta-galactosidase activity and plotted as relative luminescence units (RLU).*** p<0.001 for comparing JRFL + SLBR-N treated with mucin vs. JRFL + untreated SLBR-N by two-way ANOVA. p>0.05 for JRFL + SLBR-N treated with mannan vs. JRFL + untreated SLBR-N. Orange diamond and black triangle: RLU for JRFL infection in the presence of only mucin or mannan, respectively. **(D)** Varying effects of SLBR-N lectins on HIV-1 strains produced in primary CD4 T cells. Clade B R5- tropic or X4-tropic IMCs were produced in activated CD4 T cells from the same PBMC donor. Virions were treated with SLBR lectins, and virus infectivity was measured in TZM.bl reporter cells as in panel **(A)** To facilitate comparison across virus strains, infectivity is presented as the fold increase at the highest lectin concentration relative to untreated virus. Untreated viruses yielded RLU values ranging from 5,729 to 60,768, whereas background signals from uninfected cells ranged from 3,243 to 3,722 RLU. ****p<0.0001 **p<0.01 *p<0.05 vs. no lectin by one-way ANOVA. Comparisons with p>0.05 are left unmarked. **(E)** Different levels of virus binding to SLBR-N, SLBR-H, and SLBR-B lectins. JRFL virions were pretreated with titrating amounts of each GST-tagged SLBR lectin and then incubated with glutathione beads, and the amount of virus capture on beads was measured by RT-qPCR. Areas under the titration curves (AUC) were plotted. The blocking activity of mucin was assessed to verify O- glycan-dependent virus capture by SLBR-N. ****p<0.0001 *p<0.05 by ANOVA with Tukey's multiple comparisons test. Comparisons with p≥0.05 are left unmarked. **(F)** Varying levels of virus attachment to TZM-bl target cells in the presence of SLBR-N, SLBR-H, and SLBR-B lectins. Virus was pre-treated with titrating amounts of each lectin for 1 hour at 37°C and then incubated with TZM-bl cells for 1 hour at 4°C. The amount of cell-associated virus was quantified by RT-qPCR, and viral RNA copies were plotted.* p<0.05 for SLBR-N vs. SLBR-H and SLBR-B by two-way ANOVA with Tukey's multiple comparison. Mean ± SD are presented in panels **(A, C–F)**; data from one experiment out of two or more repeat experiments are shown. Illustrations in panels **(E, F)** were created with Biorender.com.

We subsequently measured the effect of SLBR-N on virus infectivity as measured by TCID_50_. JRFL and REJO.c IMCs were titrated, treated with SLBR-N (0.02 to 0.6 µM) for 1 h at 37°C, and then added to TZM-bl cells. SLBR-N increased virus dilutions required for 50% infection of both virus strains in a dose-dependent manner (**Figure 2B**; **Supplementary Figure 2**), confirming that this lectin indeed increased virus infectivity.

To evaluate the contribution of specific glycan–lectin interactions, we assessed the effects of mucin (Type I-S), which is richly decorated with sialylated O-glycans, and mannan, which contains high-mannose N-glycans. Mucin treatment markedly reduced SLBR-N-mediated enhancement of infectivity, whereas mannan had no discernible effect (**Figure 2C**). Neither mucin nor mannan alone altered baseline virus infectivity. These results demonstrate the role of sialylated O-glycans in SLBR-N-mediated enhancement of HIV-1 infectivity.

Because host producer cells influence virus glycan composition (Krishnamoorthy et al., 2009; Raska et al., 2014; Upadhyay et al., 2020), we next examined SLBR activity on HIV-1 virions produced in primary CD4 T cells. Eight clade B IMCs, which express *in cis* heterologous Env with X4 or R5 tropism, were propagated in activated PBMCs from a single donor. Similar to viruses produced in 293T cells, SLBR treatment enhanced infectivity of PBMC-derived viruses, although the magnitudes of enhancement varied substantially among strains (**Figure 2D**). Astonishingly, the pattern of enhancement differed by lectin. For example, infectivity of 1239-8 (X4) was most strongly enhanced by SLBR-N, followed by SLBR-B and SLBR-H, whereas 1924-H5 (X4) was unaffected by SLBR-N but showed increased infectivity in the presence of SLBR-B and SLBR-H. Interestingly, SLBR-H significantly enhanced infectivity of only X4-tropic strains and not R5-tropic strains, while SLBR-N and SLBR-B displayed activity on both X4- and R5-tropic strains. Nonetheless, all eight strains tested exhibited significant enhancement by at least one SLBR lectin. These data demonstrate differential sensitivity of virus strains to lectin-mediated enhancement of infectivity, indicating substantial heterogeneity in the abundance and composition of core 1 versus core 2 O-linked sialoglycans among HIV-1 strains.

### SLBR lectins augment HIV infectivity by binding virions and promoting virus–cell attachment

To determine whether differences in infectivity enhancement reflected variations in lectin–virion interactions, we performed virus capture assays using GST-tagged SLBR lectins. JRFL virions were pre-incubated with individual SLBR lectins and then captured by glutathione beads. Following extensive washing, lectin-bound virions captured on the beads were quantified by qRT-PCR. JRFL virions were captured most efficiently in the presence of SLBR-N, followed by SLBR-B and SLBR-H (**Figure 2E**), demonstrating that SLBR-N has greater binding capacity for JRFL than the other two lectins. Treatment with sialyated O-glycan-rich mucin significantly reduced SLBR-N-mediated virus capture, further highlighting the specific contribution of sialoglycans to HIV-1 interactions with SLBR-N.

We further examined whether lectin binding to virions facilitated virus attachment to cells. Lectin-treated JRFL virions were incubated with TZM-bl cells at 4°C for 1 h to allow virus attachment without entry. After extensive washing, cell-associated vRNA was quantified by qRT-PCR. Lectin treatment increased virus–cell attachment in a dose-dependent manner (**Figure 2F**). Notably, SLBR-N promoted higher levels of virus–cell attachment than SLBR-H or SLBR-B. This pattern closely mirrored the relative enhancement of JRFL infectivity observed in **Figure 2A**, supporting the conclusion that SLBR lectins augment virus infectivity by binding virions and promoting their attachment to target cells.

### Staphylococcal SSL lectins enhance HIV-1 infectivity in a strain- and lectin-dependent manner

We next evaluated the effect of SSL lectins on HIV infectivity. In the same DEAE dextran-free infectivity assay described above, treatment of JRFL and REJO.c with SSL3, SSL4, or SSL11 enhanced infectivity to varying levels in a lectin- and virus strain-dependent manner (**Figure 3A**). Mutations disrupting the sialic acid-binding motifs of these lectins (SSL3m, SSL4m, and SSL11m) abolished infectivity enhancement, demonstrating the requirement for glycan recognition. The enhancing activity of SSL3 was reduced by sialylated O-glycan-rich mucin but not high mannose-N-glycan-bearing mannan (**Figure 3B**), confirming a sialoglycan-dependent mechanism. Similar to SLBRs, SSL lectins also interacted directly with HIV-1 virions, and this binding was diminished by mutations at the sialic acid-binding site (**Figure 3C**). Among the three SSL lectins, SSL3 displayed the highest binding activity to both JRFL and REJO.c.

**Figure 3:**
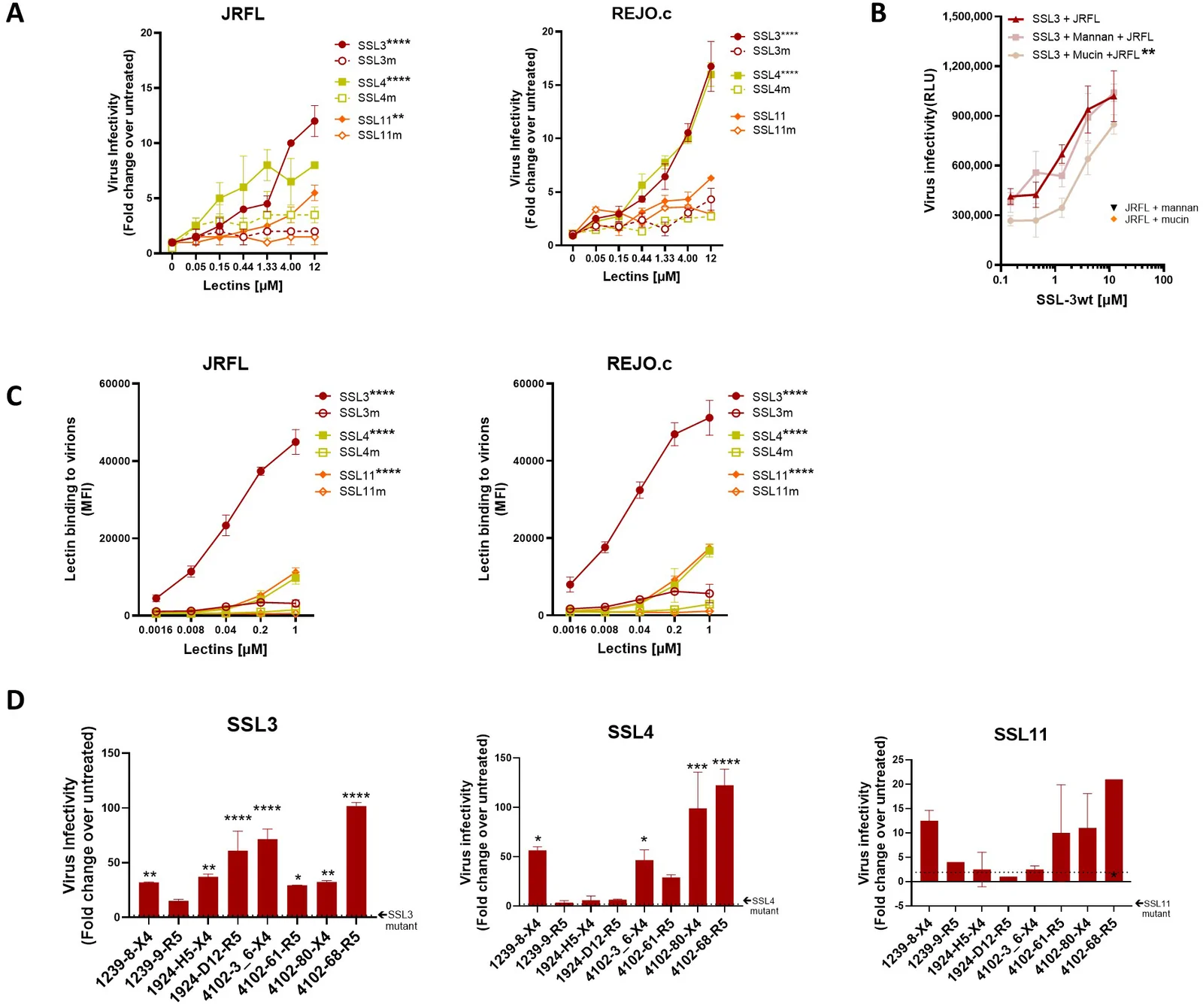
Staphylococcal superantigen-like (SSL) lectins SSL3, SSL4 and SSL11 enhance HIV-1 infectivity across different strains to varying degrees. **(A)** Enhanced infectivity of HIV-1 virions produced in 293T cells upon treatment with SSL lectins. Virions were pre-treated with titrated amounts of SSL lectins for 1 hour at 37°C and then incubated with TZM-bl reporter cells for 48 hours. Two clade B IMCs (JRFL and REJO.c) were tested with sLe^x^-binding SSL lectins and their respective mutants. Untreated virions served as controls (set to 1). Infection with untreated JRFL and REJO.C yielded 10,855 RLU and 14,202 RLU, with a background (cell alone) level of 3,012 RLU and 2,895 RLU, respectively *p<0.05, ***p<0.001, ****p<0.0001 for comparison of each SSL wt vs. its respective mutant by two-way ANOVA with Sidak's multiple comparison. **(B)** SSL3 enhancement activity sensitive to sialyated O-glycan-rich mucin and not high-mannose N-glycan-rich mannan. SSL3 was pre-incubated with 1 mg/mL mucin or mannan for 1 hour at 37°C and then incubated with JRFL for an additional 1 hour. Virus infectivity in TZM-bl reporter cells was measured 48 hours after infection. **p<0.01 for comparison of JRFL + SSL3 treated with mucin vs. JRFL + untreated SSL3 by ANOVA with Kruskal-Wallis multiple comparison test. p >0.05 for JRFL + SLBR-N treated with mannan vs. JRFL + untreated SLBR-N. Orange diamond and black triangle: RLU for JRFL infection in the presence of only mucin or mannan, respectively. **(C)** SSL bound HIV-1 virions. Virus-SSL binding was measured using a Luminex bead assay, in which virions were coupled onto beads and treated with serial dilutions of His-tagged SSL wt or mutant. SSL binding was then detected using biotinylated anti-His tag antibodies and PE-streptavidin. MFI: median fluorescence intensity. ****p<0.0001, for comparison of each SSL wt vs. its respective mutant by two-way ANOVA with Sidak's multiple comparison. **(D)** Enhanced infectivity of X4-tropic and R5-tropic clade B IMCs produced in primary CD4 T cells upon treatment with SSL3, SSL4, and SSL11 lectins (each at 12 μM). ****p<0.0001 ***p<0.001 **p<0.01 *p<0.05 vs. no lectin by ANOVA. p≥0.05 is left unmarked. Data (mean ± SD) shown in each panel were from one experiment out of two or more repeat experiments.

The SSL lectin activity was also tested using eight X4- and R5-tropic clade B IMCs produced in activated PBMCs (Registre et al., 2020). SSL3, SSL4, and SSL11 heightened infectivity of seven, four, and one virus strain, respectively, again demonstrating substantial variability among virus strains (**Figure 3D**). Overall, SSL3 and SSL4 mediated greater enhancement than SSL11, although the magnitude of enhancement was lower than that observed with SLBR-N and SLBR-B (**Figure 2D**). For all SSL lectins tested, mutation of the sialic acid-binding site abrogated the enhancing activity, confirming the dependence on lectin–glycan interactions. Together with the SLBR data, these results show that distinct bacterial lectins that recognize sialylated glycans can enhance HIV infectivity in a glycan-dependent and virus-strain-specific manner. Moreover, the greater overall enhancement observed with SLBR vs. SSL lectins indicates that binding to O-linked sialoglycans can have a more profound effect than binding to N-linked sialoglycans.

### SLBR-N, but not SSL3, enhances HIV infection in primary CD4 T cells

Because prior studies (Heindel et al., 2024) and experiments described above assessed the effects of bacterial lectins on HIV infection using TZM-bl reporter cells, we sought to examine whether similar effects were observed in primary target cells. HIV-1 reporter IMCs encoding heterologous *env* gene sequences from strains JRFL and C.1086 and expressing secreted Nano-luciferase (snNLuc) were pretreated with increasing concentrations of SLBR-N or SSL3 and then used to infect PHA-activated PBMCs or CD4 T cells obtained from two to three individual donors. Viral infection was quantified 48 h post-infection by nano-luciferase activity and expressed as fold change relative to untreated virus.

SLBR-N treatment significantly enhanced JRFL Env-mediated infection in PHA-activated PBMCs, with a consistent increase observed across donors (**Figure 4A**, top). In contrast, SSL3 exhibited no detectable effect under the same conditions. A similar pattern was observed in activated CD4 T cells, where SLBR-N treatment induced a pronounced, dose-dependent enhancement of infection, while SSL3 again failed to increase virus replication across the concentrations tested (**Figure 4B**, top). SLBR-N also increased C.1086 infection in PHA-activated PBMCs, although this activity was observed only with PBMC #6 and not with cells from the other donors (**Figures 4A, B**, bottom). SSL3 had no effect on C.1086 infection in all primary cells tested.

**Figure 4:**
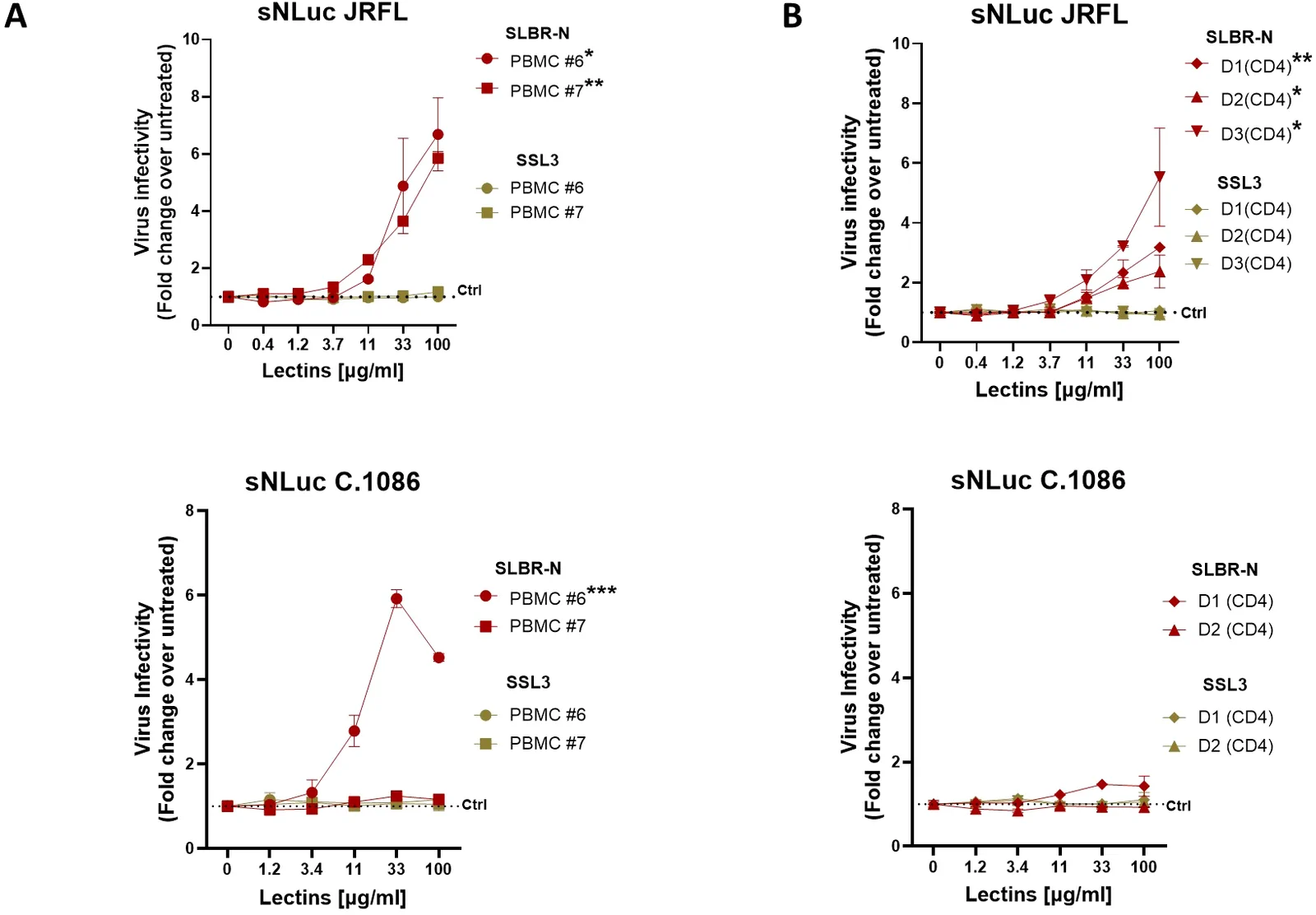
Effects of bacterial lectins SLBR-N and SSL3 on HIV-1 infection in primary CD4 T cells. JRFL and C.1086 IMC virions bearing a nanoluciferase (sNLuc) reporter gene, produced in 293T cells, were pre-incubated with titrated amounts of SLBR-N or SSL3 for 1 hour at 37°C, and added to PHA-activated PBMCs **(A)** or enriched CD4 T cells **(B)** obtained from independent donors (n=2 or 3 donors per experiment). Virus infection was measured after 48 hours using the Promega Nano-Glo Dual Luciferase Reporter Assay System. Experiments were performed in duplicate and repeated at least three times; data from one of the experiments are shown. Infection with untreated sNLuc JRFL and sNLuc C.1086 yielded mean RLU values of 2449 and 3485, respectively, whereas background signals from uninfected cells were 56 and 72, respectively. To compare across viruses, infectivity is presented as the fold changes relative to the untreated control (set to 1, dotted line). *p<0.05 **p<0.01 ***<0.001 by two-way ANOVA comparing the effects of SLBR-N vs. SSL3 on JRFL or C.1086 infection in the same PBMC or CD4 T cell donors.

These results demonstrate that SLBR-N, but not SSL3, enhances HIV-1 infection in activated PBMCs and CD4 T cells, revealing lectin-specific differences in the ability of bacterial lectins to promote HIV infectivity in primary human T-cell targets. The results also further support the importance of binding to O-linked rather than N-linked glycans for augmenting HIV infectivity.

### SLBR-N enhances HIV-1 trans-infection via DC-SIGN-independent and -dependent pathways

We next evaluated whether SLBR-N modulates HIV trans-infection. We first examined virus trans-infection from bystander cells lacking the endogenous mannose-binding C-type lectin DC-SIGN. JRFL IMC-derived virions were pre-incubated with titrated concentrations of SLBR-N and then allowed to interact with either 293T cells or RAJI cells, both of which do not express DC-SIGN. After extensive washing to remove unbound virus, these cells were co-cultured with TZM-bl target cells, and trans-infection was quantified by beta-galactosidase activity 48 h later. SLBR-N treatment increased HIV-1 trans-infection from both 293T and RAJI cells in a dose-dependent manner, with significant enhancement detected starting at 0.4 µM SLBR-N over untreated controls (**Figure 5A**).

**Figure 5:**
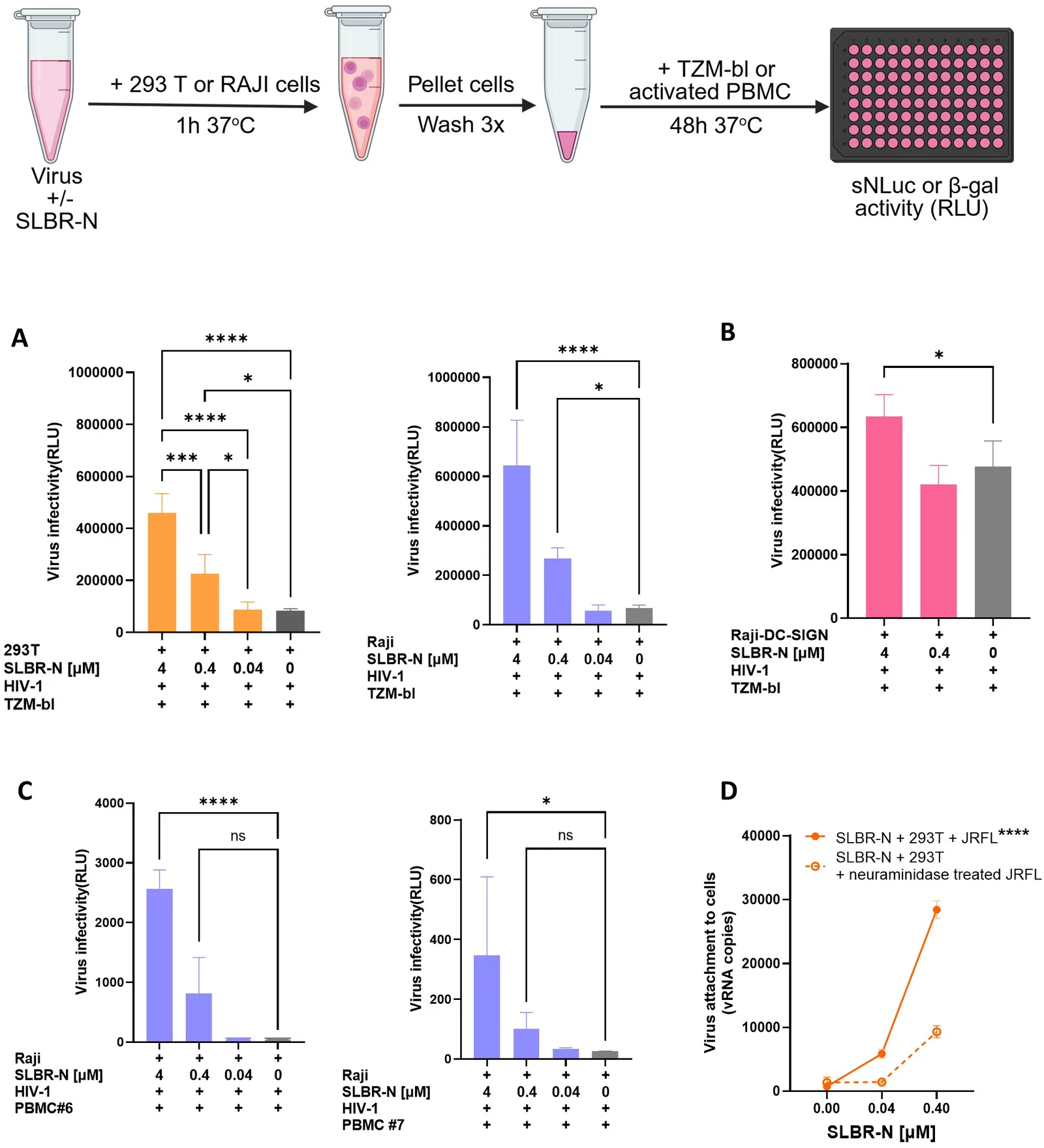
SLBR-N enhanced HIV-1 trans-infection via DC-SIGN-independent and dependent pathways. HIV-1 JRFL virions pre-treated with SLBR-N at varying concentrations were incubated with 293T and RAJI cells or DC-SIGN* RAJI cells for 1 hour at 37°C with periodic mixing. The cells were then extensively washed to remove unbound virions and co-cultured with TZM-bl cells or activated PBMCs for 48 hours at 37°C. JRFL IMC and sNLuc-expressing JRFL IMC were used in experiments with TZM-bl cells and PBMCs, respectively. **(A)** SLBR-N promoted virus trans-infection from 293T and RAJI cells to TZM-bl cells. **(B)** SLBR-N also increased virus trans-infection from DC-SIGN RAJI cells to TZM-bl cells. **(C)** SLBR-N-mediated enhancement of trans-infection was also observed from RAJI cells to activated PBMCs from donors #6 and #7. **(D)** SLBR-N enhanced virus binding to 293T cells in a dose-dependent manner. *p<0.05, ***<0.001, ****p<0.0001 by one-way ANOVA with Tukey's multiple comparisons for panels **(A–C)** ****p<0.0001 by two-way ANOVA with Sidak's multiple comparison for panel **(D)**. Experiments were performed in duplicate and repeated at least 3 times. Mean and standard error from the combined experiments are shown. p≥0.05, not marked. Illustrations were created with Biorender.com.

We then assessed the effect of SLBR-N on trans-infection from DC-SIGN^+^ RAJI cells. As expected, we observed high baseline levels of HIV-1 trans-infection attributable to DC-SIGN alone. Addition of SLBR-N further elevated trans-infection, although the magnitude of enhancement was only ~1.3-fold at the highest concentration tested (**Figure 5B**).

To determine whether DC-SIGN-independent trans-infection also occurred to primary target cells, we examined trans-infection of SLBR-N-treated sNLuc JRFL virions from RAJI cells to activated PBMCs from two independent donors. SLBR-N enhanced HIV-1 trans-infection in a dose-dependent manner to PBMCs from both donors (**Figure 5C**), consistent with the results obtained using TZM.bl target cells (**Figure 5A**).

We further investigated whether SLBR-N promoted virus attachment to bystander cells lacking DC-SIGN. Binding of HIV-1 to 293T cells increased after treatment with increasing concentrations of SLBR-N. This enhancement was markedly reduced when virions were pretreated with neuraminidase to remove sialic acid residues, indicating that SLBR-N-mediated virus–cell attachment depends on sialic acid-containing glycans (**Figure 5D**).

Collectively, these data demonstrate that SLBR-N enhances HIV-1 attachment to bystander cells lacking DC-SIGN and promotes subsequent trans-infection to permissive target cells, effectively substituting for DC-SIGN-mediated virus capture and transfer. In DC-SIGN-expressing cells, SLBR-N does not interfere with DC-SIGN function, consistent with the distinct glycan specificities of these two lectins. These findings support a model in which SLBR-N enhances HIV-1 trans-infection through both DC-SIGN-independent mechanisms and auxiliary effects in DC-SIGN-expressing cells.

### SLBR-N enhances HIV-1 infection and virus burden in humanized mice

Finally, we determined whether SLBR-N influences HIV-1 infection *in vivo* using humanized NSG (NOD scid gamma) mice. Mice (*n* = 9 per group) were derived from two independent cohorts engrafted with cells and tissues from different human donors and randomly assigned to the two groups. Mice were rectally inoculated with JRFL IMC that had been pretreated with SLBR-N or left untreated (**Figure 6A**). To model repeated mucosal exposure, mice received three sequential challenges with increasing virus doses (350, 700, and 1,400 TCID_50_). Plasma vRNA levels were monitored weekly by qRT-PCR.

**Figure 6:**
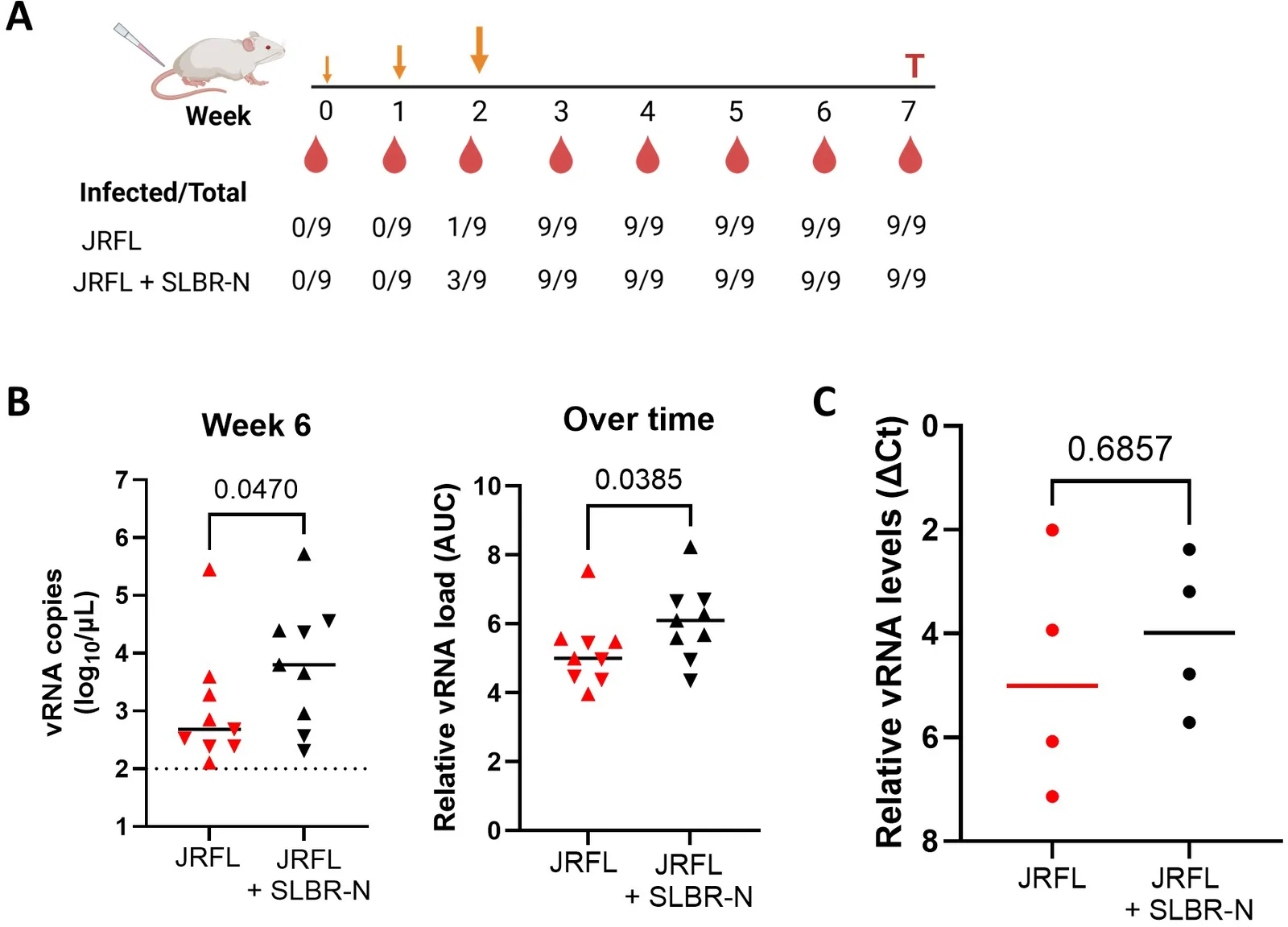
Virus burden in humanized mice after rectal inoculation of HIV-1 JRFL in the presence or absence of SLBR-N. **(A)** Experimental design. Virus was pretreated with or without SLBR-N for 1 hour at 37°C and administered rectally to humanized mice (n=9 per group) at three time points with increasing virus doses (350, 700, 1400 TCID50). Blood was collected weekly for vRNA quantification by RT-qPCR. The numbers of infected mice with vRNA copies above background are shown at the designated time points for the two groups. At the end of the study, spleen cells were collected to detect cell-associated vRNA. **(B)** vRNA load at week 6 and over time from week 2 to week 7 (as calculated by area under the curve, AUC). Two cohorts of mice generated from different human donors were assigned randomly to the two groups (up or down triangles, n=9/group). Dotted line: lower limit of detection. p values by Mann-Whitney test. **(C)** Relative vRNA levels in spleen cells from four mice from one of the cohorts at week 7 as measured by RT- qPCR. vRNA levels were calculated as ΔCt relative to GAPDH levels in the same samples. p values by Mann-Whitney test. Illustrations in panel A were created with Biorender.com.

Two weeks after the first inoculation, plasma vRNA above background was detected in one mouse in the control group, compared with three mice in the SLBR-N-treated group (**Figure 6A**). Following subsequent virus challenges, from week 3 onward, all animals in both groups became viremic. Notably, mice exposed to SLBR-N-treated virus exhibited higher overall viral burdens than controls, as evidenced by both week-6 plasma viremia and longitudinal viral loads calculated as area under the curve (AUC) (**Figure 6B**). Analysis of cell-associated vRNA in splenocytes collected at the study termination (*n* = 4 per group from the second cohort) also show a trend toward higher viral burden in mice receiving SLBR-N-treated virus (**Figure 6C**). However, substantial inter-animal variability was observed, and the difference did not reach statistical significance.

Taken together, these results show that exposure of HIV-1 to SLBR-N is associated with increased systemic viral burden following rectal inoculation, suggesting a potential role for SLBN-N in enhancing mucosal HIV-1 transmission. The study was limited by the small number of animals and variability in some virologic endpoints, although the observed trends are consistent with the *in vitro* activities of SLBR-N, including its ability to mediate virion binding, promote virus–cell attachment, and enhance virus infection and trans-infection across multiple cell types. Collectively, these findings indicate a potential role for bacterial sialic acid-binding lectins in modulating HIV-1 transmission and early infection dynamics.

## Discussion

In this study, we demonstrated using a humanized mouse model that the streptococcal sialic acid-binding lectin SLBR-N facilitated mucosal HIV-1 infection *in vivo*, as indicated by increased initial transmission and higher virus burdens after infection was established. This activity is associated with the ability of SLBR-N to bind HIV-1 virions and promote virion attachment to target cells in a sialic acid-dependent manner, resulting in enhanced infectivity in both TZM-bl reporter cells and primary CD4 T cells. SLBR-N also promoted HIV-1 capture by bystander cells and increased virus trans-infection. Although SLBR-N further augmented DC-SIGN-mediated trans-infection, prominent effects were observed in the absence of DC-SIGN, suggesting that soluble SLBR-N may facilitate trans-infection by promoting virus interactions with bystander cells, independent of CD4, CCR5 coreceptor, and DC-SIGN, although the mechanisms have not been defined. Whether SLBR-N-bound virions are internalized into bystander cells and how they are transferred to target cells remains unknown. This observation is reminiscent of prior findings with soluble host lectins. For example, soluble sialic acid-binding Siglec-7 enhances HIV-1 entry into CD4^+^ T cells and macrophages (Varchetta et al., 2013). Galectin 8 also promotes HIV-1 and HIV-2 infection by acting as a molecular bridge between virions and target cells via its dual glycan-binding domains: an N-terminal domain recognizing sialyated glycans on virions and a C-terminal domain binding to terminal galactose on CD4 T cells (Sheik-Khalil et al., 2026). Together, findings presented here substantiate and extend our prior work (Heindel et al., 2024), supporting a model in which HIV-1 virions can directly engage bacterial lectins at mucosal surfaces to enhance virus-cell attachment, augment both direct and trans-infection of CD4 T cells, and, ultimately, promote transmission efficiency.

Similar to SLBR-N, other SLBR lectins (SLBR-H and SLBR-B) and staphylococcal SSL lectins from *S. aureus* (SSL3, SSL4, and SSL11) also enhanced HIV-1 infectivity to varying extents, depending on the lectin and virus strain, although SLBR-N exhibited the greatest overall potency. The observed variability likely reflects both the fine glycan specificity of each lectin and the heterogeneity of sialoglycans among the virus strains, although the glycan compositions of the virus strains studied here have not been characterized. Env (gp120/gp41) glycosylation is highly diverse across HIV-1 strains, with many unique N- and O-linked glycosites for each strain, each of which is decorated by an array of assorted glycan structures (Silver et al., 2020; Upadhyay et al., 2020; Yang et al., 2014). Notably, changes in the Env signal sequence have been shown to alter Env glycan contents and thereby modulate Env interactions with host lectins, plant-derived antiviral lectins, and antibodies against glycan-bearing epitopes and glycan-dependent epitopes (Upadhyay et al., 2020; Yolitz et al., 2018). Consistent with this concept, we previously observed that SLBR-N reduced virus neutralization by broadly neutralizing antibodies targeting the V1V2 apex and the CD4-binding site (Heindel et al., 2024), indicating that lectin binding exerts steric or allosteric changes in Env. In addition, lectin activity was dependent on target cells, as shown by disparate effects of SSL3 in TZM-bl cells versus primary CD4 T cells. The reasons are yet unknown and need further investigation.

Notably, the magnitude of infectivity enhancement is generally lower for the SSL lectins than for the SLBR lectins. The reason for this difference remains unknown. We speculate that it may reflect differences in the interactions of SLBR and SSL lectins with HIV-1 virions. However, because the SLBR and SSL proteins carry different affinity tags, different assays were required to measure virion binding, precluding direct comparison of their binding activities. Interestingly, we also observed differences in the extent of infectivity enhancement among individual SLBRs and among individual SSLs. Although we hypothesize that lectin binding affinity contributes to these differences, virion binding did not fully correlate with infectivity enhancement. For example, SLBR-H and SLBR-B enhanced JRFL infectivity to similar levels, yet SLBR-H captured fewer JRFL virions than SLBR-B. Likewise, SSL3 and SSL4 enhanced REJO.c infectivity comparably, although SSL3 exhibited substantially greater virion binding than SSL4. These findings suggest that, in addition to virion binding, other factors contribute to lectin-mediated enhancement of HIV-1 infectivity.

It is important to note that enhancement of HIV-1 infectivity has thus far been restricted to sialic acid-binding lectins. In contrast, high mannose N-glycan-specific lectins such as *E. coli* FimH and *Lactobacillus* Msl bind HIV-1 without increasing virus infectivity. SLBR and SSL lectins tested here are specific for sialoglycan structures, such as sLe^X^ and sLn, commonly found on N- and O-glycans. Although Env is particularly rich in N-glycans, with up to 30 N-glycans per Env protomer, O-glycans have also been detected by mass spectrometry and cryo-electron microscopy, particularly in the V1 region and at the conserved C5 site near the gp120/gp14 cleavage site (Silver et al., 2020; Stansell et al., 2011; Yang et al., 2014). Computational prediction by NetOGlyc also identified potential *O*-glycan sites within the V1V2 and C5 regions (Heindel et al., 2024). While we previously showed sialic acid-dependent Env reactivity with SLBR-N, the specific SLBR-binding sites remain undefined. Virus strain-specific lectin effects observed among X4- and R5-tropic viruses sharing identical backbones, but distinct Env sequences implicate Env glycans as the primary lectin targets. However, HIV-1 virions also incorporate host-derived glycoproteins, such as MHC-I and -II (major histocompatibility complex class I and class II), LFA-1 (leukocyte-function associated antigen-1), ICAM-1 (intercellular adhesion molecule-1), CD162 (P-selectin glycoprotein ligand-1), CD43 (sialophorin), and CD44 (E-selectin ligand) (Burnie & Guzzo, 2019; Hioe et al., 1998; Murakami and Ono, 2021). These host glycoproteins carry complex N-glycans and, in many cases, O-glycans that terminate in sialic acid and thereby may play a role in lectin binding, although their contributions remain unknown.

The mechanisms by which sialic acid-binding lectins enhance HIV-1 infection are not yet fully understood. Our data demonstrate that these lectins promote virus attachment to both target cells and bystander cells independently of CD-SIGN, CD4, and coreceptors. Because each lectin contains a single sialic acid-binding site, it is unlikely that these lectins directly bridge virions to target cells. Consistent with this interpretation, we previously showed that lectin treatment of target cells did not enhance virus infectivity (Heindel et al., 2024), indicating that lectin binding to the cell surface alone is insufficient to promote infection. Instead, we propose that lectin binding to sialylated glycans on the virion surface shield their negative charge, thereby reducing electrostatic repulsion between the negatively charged viral and host cell membranes (Hu et al., 1996) (**Figure 7**). This mechanism resembles the enhancement of lentiviral infection by cationic polymers such as DEAE-dextran and polybrene, which facilitate virus–cell interactions by neutralizing electrostatic barriers (Davis et al., 2002; Denning et al., 2013; Platt et al., 2010). However, the extent of sialoglycan occupancy required to produce this effect remains to be determined. Notably, FimH and Msl lectins, which recognize uncharged mannose residues rather than sialic acid, do not enhance HIV-1 infection (Heindel et al., 2024), supporting the hypothesis that engagement of negatively charged sialoglycans, rather than lectin binding per se, underlies the observed enhancement of virus infectivity.

**Figure 7:**
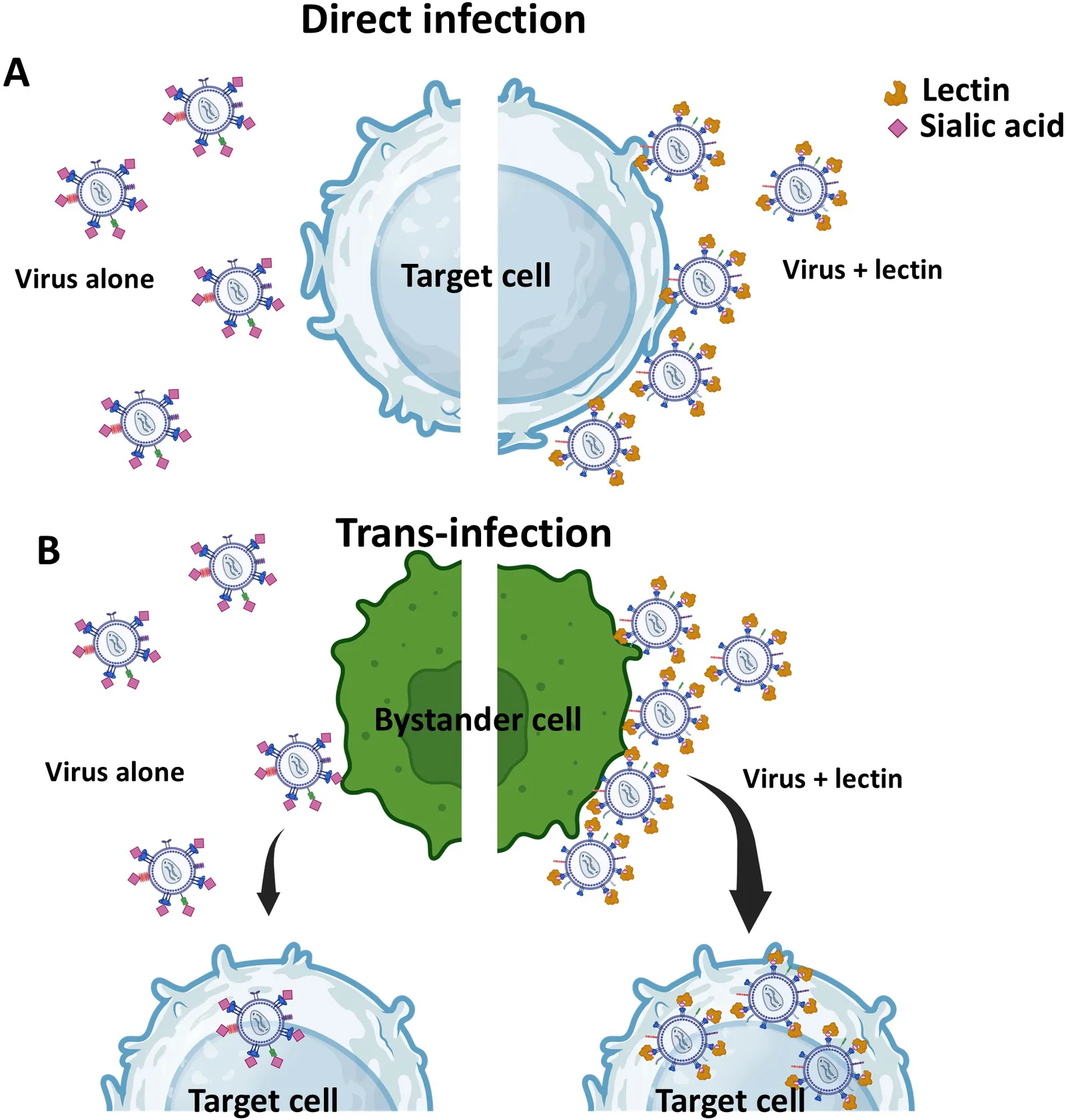
Proposed model for the mechanism by which sialic acid-binding lectins enhance HIV infection. **(A)** Direct infection. Lectins bind to sialylated glycans on Env and host-derived glycoproteins on HIV virion surface, masking negatively charged sialic acids and thereby reducing electrostatic repulsion between the negatively charged viral envelope and the host cell membrane. This enhanced proximity facilitates virus attachment and entry into susceptible target cells. **(B)** Trans-infection. Lectin binding to HIV virions also promotes virus attachment to bystander cells that do not express HIV entry receptors. Intact virions are subsequently transferred to permissive target cells, resulting in enhanced productive infection. Created with Biorender.com.

Sialic acid-binding lectins are produced by multiple genera of mucosal bacteria. These lectins often function as adhesins or virulence factors that facilitate colonization and immune evasion. For example, the *S. gordonii* SLBR lectins are expressed on the tips of fimbriae to mediate attachment to receptors such as salivary mucin MG2/MUC7 and platelet GP1ba that are implicated in oral colonization and endocarditis, respectively (Bensing et al., 2018). The SabA adhesin of *Helicobacter pylori* with phylogenetic relationship with SLBR also binds to sialylated ligands (Mahdavi et al., 2002). Similarly, *Mycoplasma genitalium* expresses a sialic acid-binding lectin called P110 or MgpC, which interacts with P140 and forms a transmembrane Nap complex on the outer bacterial surface to serve as adhesins to sialylated receptors on host cells (Aparicio et al., 2018). *M. genitalium*, which causes urethritis in men and cervicitis and pelvic inflammatory disease in women, has been associated with increased HIV-1 risk (Mavedzenge et al., 2012; Smullin et al., 2020). In comparison, staphylococcal SSL lectins are secreted proteins that downmodulate immune responses by interacting with myeloid cells, inhibiting TLR activation, or preventing IgA binding to FcαRI on neutrophils (Chung et al., 2007; Hermans et al., 2012; Koymans et al., 2015, 2017). Despite these precedents, the prevalence, regulation, and functional impact of sialoglycan-binding lectins within the mucosal microbiota remain largely unexplored. Importantly, their contribution to HIV-1 transmission is likely context dependent, influenced by factors such as the mucosal site, local microenvironment, and host factors. These variables influence not only the abundance and diversity of lectin-producing bacteria but also the kinetics and magnitude of lectin expression, which may ultimately determine the extent to which sialic acid-binding bacterial lectins and possibly other microbial lectins modulate HIV-1 infectivity following mucosal exposure.

These findings may have particular relevance in clinical scenarios in which medical interventions perturb the mucosal microbiota, potentially modulating HIV-1 risk. Genital secretions from Black and Hispanic women who received Depo Provera enhanced HIV-1 infectivity *ex vivo* (Tasker et al., 2025; Yang et al., 2019). In this population, Depo Provera administration was associated with disruption of the vaginal bacterial network, signified by loss of *Lactobacillus* connections and persistence of those with *Streptococcus*, *Mycoplasma*, and a limited number of other species (Yang et al., 2019). Given the documented association between Depo Provera use and increased risk of HIV-1 acquisition (Brind et al., 2015; Hapgood et al., 2018; Morrison et al., 2015; Polis et al., 2016; Ralph et al., 2015), these observations underscore the need to investigate further whether bacterial lectins contribute to the heightened virus susceptibility under such conditions.

## Conclusion

This study identifies sialic acid-binding lectins from common bacterial species of the human microbiota as potential modulators of HIV-1 transmission. These lectins interact directly with HIV-1 virions in a glycan-specific manner, promoting virus attachment to host cells and increasing virus infectivity through both direct infection and trans-infection pathways. Moreover, the presence of these lectins promotes HIV-1 infection in the context of mucosal transmission in a humanized mouse model. Collectively, these findings provide new insights into how the human microbiota may influence HIV-1 transmission across mucosal surfaces and underscore the importance of microbial–viral interactions as contributors to HIV-1 acquisition and pathogenesis.

## Limitations

This study primarily utilized recombinant bacterial lectins rather than native lectins expressed on streptococcal fimbriae and secreted by staphylococcal species. While this reductionist approach enabled attribution of virus infectivity-enhancing activity specifically to the lectins, additional studies are needed to validate these findings in the context of intact bacteria, polymicrobial communities, and mucosal fluids, where diverse host and microbial factors may influence virus–lectin interactions. For example, rectal HIV-1 challenge studies in humanized mice could be performed in the presence of isogenic *S. gordonii* strains that differ only in expression of SLBR-N. Alternatively, infectivity assays could be conducted in vaginal or other genital secretions containing defined levels of endogenous bacterial lectins.

Another limitation is that the physiological concentrations of bacterial lectins and sialic acid-binding lectins specifically at mucosal surfaces remain unknown. To our knowledge, quantitative measurements of bacterial lectins *in vivo* have not been reported. Although *S. aureus* can be detected in vaginal secretions at mean concentrations of 10^3.7^ cfu/mL (Wilks et al., 1984), the levels of secreted SSL lectins in these samples have not been determined. Likewise, *Streptococcus*, *Staphylococcus*, and *Pseudomonas* species can achieve high local densities within biofilms (10^8^–10^9^ bacteria/μL) (Hou et al., 2019), creating microenvironments that may contain substantial concentrations of surface-associated or secreted lectins. Given that individual streptococcal cells may express hundreds to thousands of lectin-capped fimbriae, localized lectin concentrations at such mucosal microenvironments could approach the range evaluated in our experiments. For example, 0.10 μM of SLBR is equivalent to ~6 × 10^10^ molecules/μL, a concentration achievable with 10^8^ bacteria/μL. Moreover, the positioning of SLBR lectins at the distal tips of flexible fimbriae is expected to facilitate access to virions, in the same way as that to glycans on host cells. Future studies that directly quantify lectin abundance in mucosal secretions and biofilms will therefore be important for defining the physiological relevance of the concentrations tested here. Importantly, sialic acid-binding lectins are produced by multiple bacteria species, and their cumulative production within dynamic microbial communities, particularly during perturbations in microbial composition, inflammation, and hormonal changes, could influence HIV-1 infectivity at mucosal sites.

We have provided initial evidence that bacterial lectins can facilitate HIV-1 trans-infection independently of the host lectin DC-SIGN. Although the use of 293T and RAJI cell lines allowed clear discrimination of trans-infection mediated by bacterial lectins vs. DC-SIGN, these systems do not fully reflect physiologic conditions. Further studies should incorporate primary cell types, including vaginal, cervical, and foreskin epithelial cells, as well as dendritic cells expressing DC-SIGN, Siglecs, and other host lectins.

Finally, experiments using primary PBMCs and CD4 T cells were performed with cells from a limited number of donors (*n* = 2–3) and larger donor cohorts will be needed to evaluate inter-individual variability in susceptibility to lectin-mediated enhancement of HIV-1 infection. Likewise, the *in vivo* experiments were conducted in a limited number of humanized mice derived from two different donor cohorts. Although consistent results were observed with two cohorts, this model has inherent limitations: it does not fully recapitulate the structural and functional characteristics of human mucosal tissues; its NSG background lacks B-cell and antibody repertoires and precludes the establishment and maintenance of a representative human mucosal microbiota (Chupp et al., 2024); and HIV-1 infection was achieved using high-dose rectal challenges that do not reflect the relatively low efficiency of sexual transmission in humans (Patel et al., 2014). Accordingly, the lectin-mediated enhancement observed in this model should be validated in additional preclinical models and, ultimately, in human studies.

## Data Availability

The original contributions presented in the study are included in the article/**Supplementary Material**. Further inquiries can be directed to the corresponding author.
